# 造血干细胞移植后肺部慢性移植物抗宿主病的诊疗策略

**DOI:** 10.3760/cma.j.cn121090-20250128-00048

**Published:** 2025-08

**Authors:** 春蓉 巨, 顺清 王, 曦 张, 启发 刘

**Affiliations:** 1 广州医科大学附属第一医院器官移植科，广州呼吸健康研究院，国家呼吸医学中心 国家呼吸系统疾病临床医学研究中心 呼吸疾病国家重点实验室，广州 510120 Department of Organ Transplantation, the First Affiliated Hospital of Guangzhou Medical University, Guangzhou Institute of Respiratory Health, National Center for Respiratory Medicine, National Center for Clinical Medical Research of Respiratory Diseases, State Key Laboratory of Respiratory Diseases, Guangzhou, 510120, China; 2 广州市第一人民医院血液内科，广州 510180 Guangzhou First People's Hospital, Guangzhou, 510180, China; 3 陆军军医大学第二附属医院血液病医学中心，重庆 401147 Hematology Medical Center, The Second Affiliated Hospital of Army Medical University, Chongqing, 401147, China; 4 南方医科大学血液病研究院，广州 510515 Institute of Hematology, Southern Medical University, Guangzhou 510515, China

## Abstract

造血干细胞移植（HSCT）是目前根治恶性血液病及骨髓衰竭性疾病的有效手段，已经被普遍应用。近年来我国的HSCT迅速发展，但移植后肺部慢性移植物抗宿主病却严重影响患者生活质量和远期生存率。本文针对肺部慢性移植物抗宿主病的高危因素、临床分型及早期诊断策略进行了简单述评，同时对目前的药物治疗方案进行了详细分类评述，并提出在肺部cGVHD终末期内科治疗无效时，肺移植是唯一而有效的治疗手段。

## Abstract (en)

Hematopoietic stem cell transplantation（HSCT）is currently an effective method to cure hematologic malignancies and bone marrow failure diseases, and can restore hematopoietic function destroyed by hematologic malignant diseases. In recent years, HSCT has developed rapidly in China, but pulmonary chronic graft-versus-host disease after transplantation has seriously affected the quality of life and long-term survival of patients. Therefore, this article aims to describe the risk factors, clinical classification, and early diagnosis and treatment strategies of pulmonary chronic graft-versus-host disease, and proposes that lung transplantation is the only effective therapeutic intervention when medical treatment proves ineffective for end-stage pulmonary cGVHD.

造血干细胞移植（hematopoietic stem cell transplantation, HSCT）作为血液系统恶性疾病重要的根治手段，可恢复已被严重破坏的造血功能，目前已经广泛应用于临床。按照供者来源，HSCT可以分为自体造血干细胞移植（auto-HSCT）和异基因造血干细胞移植（allo-HSCT）。目前越来越多的血液系统恶性疾病患者可接受allo-HSCT，全球77个国家每年进行超过68 000次HSCT[1]–[2]。尽管allo-HSCT的数量迅速增加，但术后并发症仍然是血液科专家面临的一大挑战。这些并发症可能影响皮肤、胃肠道、肝脏以及肺脏等多个器官系统，其中，肺脏作为HSCT后并发症的主要受累器官之一，不仅严重影响患者的生活质量，而且影响患者的早期和远期存活率。

HSCT后的肺部并发症包括感染性并发症和非感染性并发症。感染性并发症主要以免疫抑制宿主性肺炎为代表，按照病原体可分为细菌性肺炎、侵袭性肺真菌病、病毒性肺炎、非典型病原体肺炎等。而非感染性并发症，按照移植后时间可分为移植后早期肺部并发症和中远期并发症，前者主要包括围植入期呼吸窘迫综合征（peri-engraftment respiratory distress syndrome, PERDS）、弥漫性肺泡出血（diffuse alveolar haemorrhage, DAH）、特发性肺炎综合征（idiopathic pneumonia syndrome, IPS）、肺动脉高压（pulmonary aterial hypertension, PAH）等。早期的非感染性肺部并发症发生率相对较低，而远期迟发性非感染性肺部并发症（delayed non-infectious pulmonary complications, LONIPC）发生率较高，是影响患者生活质量和远期生存率的主要并发症之一。其中，慢性移植物抗宿主病（cGVHD）对肺部的损害是最为棘手的问题之一。针对肺部cGVHD对肺功能造成损害后出现严重呼吸衰竭、内科药物治疗无效的终末期患者，肺移植是唯一有效的治疗手段。

一、肺部cGVHD的分型

目前，以阻塞性通气功能障碍为主要表现的闭塞性细支气管炎综合征（bronchiolitis obliterans syndrome, BOS）是被公认的肺部cGVHD的典型表现，也最为常见，而以限制性通气功能障碍为主要表现的间质性肺病（interstitial lung disease, ILD）发生率虽然较低，但与BOS相比，其进展更迅速、预后更差，因此，近年来越来越受到关注。cGVHD相关BOS（cGVHD-BOS）经常发生于HSCT后100 d至2年间，但也有患者发生于HSCT后5~6年，且往往发生在活动性cGVHD再次出现时[3]–[5]。cGVHD-ILD可以表现为多种不同的类型，包括机化性肺炎（organizing pneumonia, OP）、非特异性间质性肺炎（nonspecific interstitial pneumonia, NSIP）、弥漫性肺泡损伤（diffuse alveolar damage, DAD）、淋巴细胞间质性肺炎（lymphocytic interstitial pneumonia, LIP）和另外一种特殊的亚型-胸膜肺弹性纤维样变（pleural pulmonary elastic fibrosis, PPFE），PPFE会导致严重的限制性通气功能障碍，病理改变以胸膜和肺的纤维化为主，也被归于ILD。

二、肺部cGVHD的高危因素

cGVHD-BOS的危险因素包括：HSCT早期采用包含白消安的清髓方案，巨细胞病毒（CMV）血清IgG阳性，移植前存在基础肺疾病，女性供者，无关供者，以及既往存在急性GVHD（aGVHD）病史。HSCT的供患者之间主要组织相容性复合体或次要组织相容性抗原不匹配引起的免疫激活是cGVHD的主要危险因素。过去认为，在HLA全相合移植的患者中，cGVHD的发生率相对较低，然而，通过对国家呼吸医学中心收治的病例总结发现，在肺部cGVHD呼吸衰竭同时接受肺移植的患者中，HLA全相合移植患者占比更高[6]。分析其中的原因，可能是全相合移植患者移植后早期免疫抑制强度较低，反而导致后期肺部cGVHD及呼吸衰竭的发生率更高、病情也更严重。

呼吸道感染（包括细菌、真菌、病毒及非典型病原体感染）也是诱发肺部cGVHD的高危因素，病毒感染诱发的BO（post-infection bronchiolitis obliterans, PIBO）较为常见。对于儿童，腺病毒、呼吸道合胞病毒、肺炎支原体等为PIBO的常见病原体；而在HSCT患者中，CMV是最常见的BOS病原体。CMV感染后，不仅通过直接因素损伤靶器官，而且通过调控免疫活性细胞引发炎症反应，从而诱发肺部GVHD[7]–[8]。也有研究表明：患者年龄较大、HSCT前存在气流阻塞也是cGVHD-BOS的风险因素[9]。

既往有aGVHD病史、胃食管反流、微误吸、空气污染、既往胸部放疗等高危因素，在病理生理机制方面，涉及多种免疫细胞、细胞因子、趋化因子和生长因子等多层面的相互作用[10]。

三、肺部cGVHD的临床特征

1. 临床表现：肺部cGVHD早期一般没有任何症状或仅有轻微干咳，隐匿发生，随着疾病进展，开始出现活动耐量下降，逐渐进展为活动后胸闷、气促，疾病显著进展，出现气促甚至呼吸困难。查体的体征方面，肺部cGVHD-BOS患者会逐渐出现桶状胸、呼吸音减弱，合并支气管扩张者可闻及肺部湿啰音；而肺部cGVHD-ILD患者的肺部听诊可闻及velcro啰音。

2. 肺部影像学表现：肺部cGVHD-BOS典型的影像学表现为双肺透亮度增加、双肺容积显著增大、肺过度充气、局部支气管扩张、支气管壁增厚、空气潴留征、马赛克征；其中，肺过度充气征、空气潴留征、马赛克征被称为cGVHD-BOS三联征。部分患者出现气胸、纵隔气肿等影像学表现，多见于严重BOS病例。临床上，出现上述典型的影像学表现时，对应的肺功能已经严重下降。在疾病早期，通过薄层胸部CT进行吸气末和呼气末两个时相的扫描对比，就可以发现气体陷闭阳性，为BOS早期影像学表现。肺部cGVHD-ILD的诊断，即高分辨率CT（HRCT）上存在浸润性混浊[11]。影像学表现为双肺多发的圆形或不规则斑点状高密度影、肺实质纤维化、肺内网状影、胸膜下线影、肺泡壁增厚等。cGVHD-BOS、cGVHD-ILD及PPFE的典型影像学表现见图1。

**图1:**
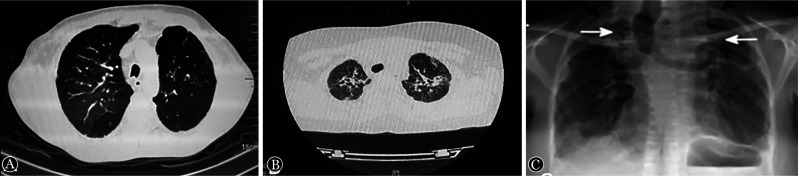
慢性移植物抗宿主病相关闭塞性细支气管炎综合征（BOS）、间质性肺病（ILD）及胸膜肺弹性纤维样变（PPFE）的典型影像学表现 **A** BOS影像学表现（肺容积显著增大、支气管管壁增厚，存在空气潴留征、马赛克征、肺过度充气征）；**B** ILD影像学表现（双肺多发圆形或不规则斑点状高密度影、肺实质纤维化、肺内网状影、胸膜下线影、肺泡壁增厚）；**C** PPFE影像学表现（以肺尖为主的胸膜下区域纤维化）

3. 肺组织病理学：肺部cGVHD引发的BOS和ILD表现不同。BOS的组织损伤部位主要在小气道、病理表现为小气道闭塞；而ILD的组织损伤部位主要在肺间质，表现为间质性肺炎或肺纤维化。BOS的病理生理过程为小气道上皮细胞和亚上皮结构的炎症反应，组织损伤和过度的纤维化增生，最终引发气道闭塞[12]。ILD主要病理表现为显著的间质性肺炎、肺组织结构重塑导致的肺纤维化，以肺泡间质纤维化伴淋巴细胞性炎症、肺纤维化和小叶间隔增厚为特征[10]。其中，PPFE作为ILD的一种特殊类型，主要表现胸膜组织异常增厚、胸膜组织和靠近胸膜的肺组织发生弹性纤维样变性，上肺为好发部位。

四、肺部cGVHD的肺功能监测及诊断

无论是哪一种类型的肺部cGVHD，对肺功能的损害通常起病隐匿，早期无症状，但出现明显症状时肺功能已严重受损。因此，对于allo-HSCT患者，无论在接受移植时与供者的HLA配型是否相合，均应从移植术后早期就关注患者的肺功能，密切地监测肺功能，建议所有allo-HSCT患者进行居家肺功能仪检测，每周至少1次。有研究表明，通过简易肺功能仪动态监测，可以准确反应肺功能变化趋势[13]。如简易肺功能结果提示FEV1下降>10％持续2周以上，需立即就医，明确肺功能下降的原因，建议每3个月至正规医疗机构进行1次完整的肺功能检测[14]。

临床上，当患者已经出现胸闷、气促、甚至呼吸衰竭的时候才意识到肺部cGVHD，已经为时过晚。因此，对于allo-HSCT后的患者，肺功能的定期监测非常重要，肺cGVHD的BOS与ILD分型诊断详见表1。BOS的诊断主要根据患者进行性加重的呼吸困难的临床表现、肺功能检测及胸部CT结果，cGVHD-BOS的肺功能分级诊断详见表2。肺部cGVHD-ILD的诊断[11]，即HRCT上存在浸润性混浊，并且：①呼吸道样本（BALF、鼻腔吸出物、痰液）中未发现病原体；②尽管进行了广泛的抗感染治疗，但没有观察到临床或影像学改善；③在肺活检上没有发现病原体（如果有条件进行肺活检）。

**表1:** 肺部慢性移植物抗宿主病相关BOS与ILD表现

疾病	FEV1/FVC < 0.7	TLC相较于基线下降 ≥ 10％	CT影像渗出表现
BOS	是	否	否
ILD	否	是	是

**注** BOS：闭塞性细支气管炎综合征；ILD：间质性肺病；FEV1/FVC：1秒率；TLC：肺总量

**表2:** 慢性移植物抗宿主病相关BOS的肺功能分级诊断标准

分级	FEV1/FVC值	FEV1值
BOS0	FEV1/FVC<70％	FEV1≥80％pred
BOS1	FEV1/FVC<70％	FEV1=60％~79％pred
BOS2	FEV1/FVC<70％	FEV1=40％~59％pred
BOS3	FEV1/FVC<70％	FEV1≤39％pred

**注** BOS：闭塞性细支气管炎综合征；FEV1/FVC：第一秒所呼出的气容积占总呼气容积的百分比；FEV1：最大深吸气后，做最大呼气第一秒所呼出的呼出气容积

五、肺部cGVHD的治疗

目前，对于确诊的HSCT后的肺部cGVHD，尚缺乏广泛认可的统一治疗方案。以下就BOS的对症支持治疗、抗炎治疗、抗纤维化治疗等进行论述。推荐治疗流程图见图2。

**图2:**
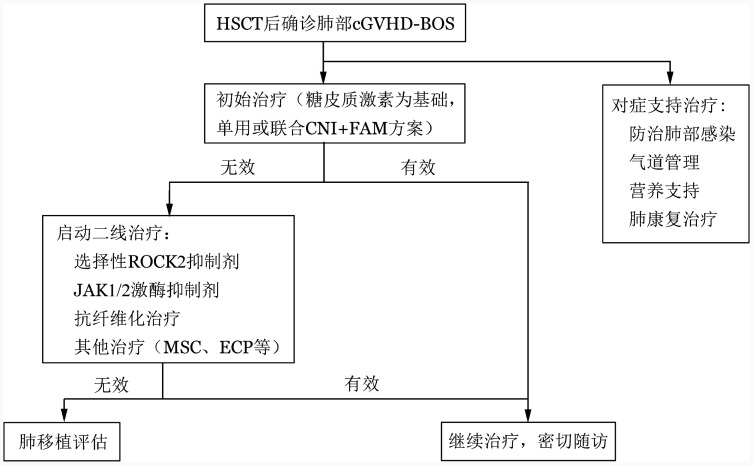
闭塞性细支气管炎综合征（BOS）治疗流程图 **注** CNI：钙调磷酸酶抑制剂；FAM：氟替卡松+阿奇霉素+孟鲁司特

（一）对症支持治疗

1. 预防和治疗肺部感染：移植患者由于长期使用免疫抑制剂，免疫功能低下，容易发生肺部感染。而多种病原体（包括细菌、真菌、病毒、非典型致病菌等）感染，均可能诱发肺部cGVHD，导致肺功能进行性下降。其中，CMV感染后诱发肺部cGVHD-BOS较常见，因此，临床需要高度关注HSCT后的CMV感染，尤其是难治性或耐药CMV感染[7]。对于CMV感染的高危人群，建议采用更昔洛韦/缬更昔洛韦或来特莫韦予以预防；无法耐受长期普遍预防的患者，应该严密监测血CMV-DNA并优化用药方案，可以采用抢先治疗策略，药物选择包括静脉用药更昔洛韦、口服的缬更昔洛韦或来特莫韦、膦甲酸钠、西多福韦等；对于难治性CMV感染患者，可以采用马立巴韦等[8],[15]，同时定期监测血液CMV-DNA[16]。另外，由于HSCT患者存在全身免疫功能及局部黏膜免疫功能低下，也容易反复发生呼吸道细菌感染。在肺部cGVHD发展至疾病后期，部分患者由于反复继发肺部感染等因素，常伴有肺结构破坏及广泛支气管扩张，痰液引流困难，抗感染治疗效果不佳。针对这些患者，在积极抗感染治疗的同时，应该加强气道管理，促进痰液排出，减轻气道炎症反应及气道壁黏膜水肿。而且，肺结构破坏及支气管扩张容易合并存在耐药细菌定植或真菌定植，进一步加重肺功能慢性损害，因此，可以选用针对性抗生素或抗真菌药物进行雾化吸入，予以去定植治疗。铜绿假单胞菌定植，可以选择雾化吸入的抗生素如妥布霉素吸入剂、多粘菌素吸入剂、阿米卡星脂质体吸入剂等，而曲霉或念珠菌等真菌的定植，可以考虑吸入两性霉素B[17]。

2. 营养支持及肺康复治疗：HSCT患者由于存在不同程度的肠道cGVHD或受到药物的影响，往往存在胃肠道黏膜受损，胃肠道吸收功能障碍，因此，多数患者存在营养不良、低体重及器官功能衰竭[18]。肠内营养可提高总生存率、减少血小板移植时间、降低急性胃肠道cGVHD发生率及缩短平均住院时间，已经被美国肠外与肠内营养学会及欧洲临床营养代谢协会推荐作为这类患者营养支持的一线选择[19]。有效的营养支持配合呼吸康复治疗能有效减少呼吸道分泌物潴留，促进痰液排出，减少呼吸道炎症因子对气道的反复刺激及支气管重塑的发生，同时可辅助肺复张，促进呼吸功能恢复，调节由于过度充气引起的肺组织过张，缓解肺部cGVHD-BOS患者呼吸困难的主观症状，改善其运动耐量[20]。

（二）抗炎治疗

1. 糖皮质激素：BOS是目前公认的HSCT后肺部cGVHD的典型表现[21]。目前，肺部cGVHD的一线治疗方案为糖皮质激素联合或不联合钙调磷酸酶抑制剂（calcineurin inhibitors, CNI）类药物，CNI类药物包括环孢素A（CsA）和他克莫司。糖皮质激素作为肺部cGVHD的一线治疗方案[22]，常规治疗剂量为泼尼松1 mg·kg^−1^·d^−1^，但长期应用糖皮质激素不良反应多，而且，对BOS效果不明显[23]，目前已较少单独长期应用于HSCT后BOS治疗。发生糖皮质激素难治或依赖时，可选用二线药物如甲磺酸贝舒地尔、JAK1/2激酶抑制剂、酪氨酸激酶抑制剂、利妥昔单抗、钙调神经磷酸酶抑制剂和霉酚酸酯[24]。针对LONIPC的治疗，需要根据病因制定相应的治疗策略；由免疫排异反应导致的肺损伤，如果无合并感染，建议尽早给予免疫抑制剂治疗（包括BOS和ILD），诊断后早期（7 d内）启动糖皮质激素治疗（等效泼尼松≥ 1 mg·kg^−1^·d^−1^），一个月内减量，可以改善患者的长期生存[25]–[26]。但是，针对感染后ILD，确保感染得到有效控制的前提下，可予以糖皮质激素等免疫抑制剂抑制炎症反应，同时启动抗纤维化治疗。

2. CNI类药物：CsA和他克莫司均属于CNI类药物；作为长期维持的免疫抑制剂，广泛应用于实体器官移植和HSCT患者。他克莫司的免疫抑制作用较CsA更强，因此，针对肺部cGVHD患者，如果使用CsA治疗效果不理想，建议切换为他克莫司。CsA和他克莫司均有较严重的肾毒性、心血管系统及血糖血脂等代谢异常的不良反应，他克莫司对糖代谢的影响更大，因此，针对有糖尿病或严重糖耐量异常的患者，需谨慎切换。和口服用药相比，局部雾化吸入药物可直接在免疫激活部位发挥免疫抑制作用，减少全身暴露，有效缓解药物的不良反应。研究表明，CsA脂质体雾化吸入可改善或稳定肺功能参数，在肺移植患者中显示出较好的疗效[27]。对于肺部cGVHD-BOS患者，目前已有临床试验正在探究CsA脂质体吸入剂替代口服CsA，作为常规免疫抑制剂应用于HSCT后cGVHD-BOS患者。

3. JAK1/2激酶抑制剂：芦可替尼（ruxolitinib）可抑制效应性T细胞和促炎细胞因子的生成[28]，被作为HSCT后肺部cGVHD的二线治疗用药。JAK1/2激酶抑制剂已逐渐应用于对糖皮质激素不耐受的肺部cGVHD患者。芦可替尼可以降低部分肺部cGVHD-BOS患者糖皮质激素需求量，并有助于稳定肺功能、延缓肺功能下降速度[29]。然而，芦可替尼对肺部cGVHD的应答率及缓解率较低，且存在诱发感染和CMV再激活问题[30]。STAT抑制剂伊马替尼治疗cGVHD的6个月应答率为67％，但主要针对皮肤黏膜的应答率较高，对肺部cGVHD的应答率不足10％，而且存在诱发肺炎等感染问题；伊布替尼对皮肤型cGVHD缓解率较高，但同样存在诱发肺炎和真菌感染问题[31]。总体来说，JAK1/2激酶抑制剂对肺部cGVHD治疗效果不理想。

4. 阿奇霉素：阿奇霉素为半合成的十五元环大环内酯类抗生素，具有抗炎效果及免疫调节功能，已广泛应用于肺移植术后慢性排斥反应相关BOS的治疗。肺移植后慢性排斥反应导致BOS与HSCT后肺部cGVHD相关BOS的发病机理类似，均是由于免疫细胞介导的慢性持续性炎症反应对小气道的损害。阿奇霉素是肺移植领域目前为止研究最多、最成熟的BOS治疗药物。部分移植中心建议肺移植患者终身小剂量（250 mg隔日1次）服用阿奇霉素以预防BOS。然而，阿奇霉素在HSCT后的BOS患者中的具体作用及不良反应仍存在较大争议。Bergeron等[32]研究发现，预防性应用阿奇霉素并不能有效降低HSCT后BOS发生率，而且，后期分析发现阿奇霉素组血液病复发率（33.5％）高于安慰剂组（22.3％），提示其可能增加复发风险。Cheng等[33]研究显示，阿奇霉素与新发恶性肿瘤的风险增加相关，但与原始血液恶性肿瘤复发的风险无关。阿奇霉素对BOS患者的利弊需要进一步研究证实。

5. 联合气道雾化吸入糖皮质激素方案：欧洲血液和骨髓移植协会推荐将氟替卡松（fluticasone）吸入、阿奇霉素及孟鲁司特（montelukast）口服的FAM方案联合糖皮质激素治疗，并在1个月内糖皮质激素迅速减量作为HSCT后cGVHD-BOS患者的初始治疗方案[23]。其中，吸入氟替卡松可减轻局部炎症反应，阿奇霉素减少白细胞介素-8水平和中性粒细胞聚集，孟鲁司特通过白三烯途径抑制炎症细胞的增殖和黏附，FAM方案目前已经被多个中心采纳来治疗cGVHD-BOS，能够有效减少糖皮质激素的应用剂量，降低糖皮质激素相关的不良反应。类似的PMN方案，其中包括普米克（pulmicort）吸入、孟鲁司特和N乙酰半胱氨酸（N-acetylcysteine）口服。然而，并非所有的cGVHD-BOS患者对FAM或PMN方案治疗有反应，而且，长期糖皮质激素吸入还有引发气道真菌感染的风险，值得关注并谨慎选择使用人群及疗程。

（三）抗纤维化治疗

吡非尼酮和尼达尼布均属于临床用于治疗间质性肺疾病特异性的靶向抗肺纤维化药物，能抑制成纤维细胞增殖和胶原合成。

1. 吡啶酮类：BOS的特点是肿瘤生长因子TGF-β驱动的支气管周围炎症、细胞外基质沉积和纤维化导致的支气管狭窄[34]。吡非尼酮是一种新的具有广谱抗纤维化作用的吡啶酮类化合物，可减弱TGF-β刺激的胶原合成，减少成纤维细胞增殖，已被批准用于特发性肺纤维化。吡非尼酮抑制肺支气管发生结构性变化，缓慢抑制肺功能恶化及纤维化进程[35]。Matthaiou等[36]对30例HSCT后BOS患者应用吡非尼酮推荐剂量2 403 mg/d治疗，结果显示，38％的参与者在治疗期间FEV1％和FEV1/FVC％轨迹有所改善，提示吡非尼酮可抑制肺功能恶化。但是该研究仅证实了患者可耐受推荐剂量的吡非尼酮，缺乏对吡非尼酮应用疗效的长期评定。基于HSCT后cGVHD-ILD的病理特征，也可考虑使用。

2. 酪氨酸激酶抑制剂（TKI）：尼达尼布（nintedanib）是一种具有多个靶点的TKI，通过抑制血小板衍生的生长因子途径发挥抗纤维化作用，目前已被批准用于特发性肺纤维化的治疗，但对于HSCT后肺部cGVHD-BOS的治疗目前尚缺乏临床证据。曾有研究者报道1例18岁HSCT后BOS患者应用尼达尼布的疗效观察，该患者FAM方案联合糖皮质激素治疗效果欠佳的情况下，保留原免疫抑制治疗方案并加用尼达尼布，患者症状快速缓解且PFT出现改善，提示尼达尼布可作为一种新的治疗选择[37]。伊马替尼（imatinib）是一种高选择性的小分子TKI，通过阻断TGF-β或PDGF信号通路的传导，抑制纤维化进展。伊马替尼治疗肺部cGVHD-BOS患者的有效率为76.9％[29]。

然而，目前尚无高质量的循证医学证据证明抗纤维化药物在HSCT后肺cGVHD中的作用，对于启动抗纤维化治疗的时机仍需探讨。建议在影像学提示纤维化进展或肺功能快速下降时给予抗纤维化治疗。

（四）抗炎及抗纤维化治疗

甲磺酸贝舒地尔（belumosudil mesylate）是一种口服的选择性ROCK2抑制剂，通过调节STAT3/STAT5磷酸化，下调过度活化的Th17，增强调节性T细胞功能，进而抑制促炎反应，重建免疫稳态；另外，甲磺酸贝舒地尔可以通过抑制TGF-β抑细胞增殖而逆转纤维化，具有抗炎和抗纤维化双重作用机制，可在重建免疫稳态的同时下调纤维化过程[38]。针对甲磺酸贝舒地尔治疗肺部cGVHD-BOS的前瞻性研究结果显示，该药对于临床上难治性肺部cGVHD有显著疗效。根据NIH应答标准，入组患者最佳缓解率为32％，完全缓解率可达15％[39]。在美国，甲磺酸贝舒地尔片已经于2021年7月获批上市，主要用于对HSCT后GVHD、既往接受至少2线系统性治疗失败的肺部cGVHD。甲磺酸贝舒地尔作为抗炎抗纤维化的药物，已经在全球完成了Ⅲ期临床研究，前期的研究结果显示：该药物能够显著改善肺部cGVHD患者的肺功能，提高其生活质量、延长生存期。因此，甲磺酸贝舒地尔片于2023年8月获批用于治疗对糖皮质激素或其他系统治疗应答不充分的cGVHD患者。前期研究显示，针对cGVHD患者，甲磺酸贝舒地尔在肺部应答率可以达到26％，显著高于中国获批的另外一种治疗cGVHD的二线用药芦可替尼（8.2％）；该药目前是中国唯一获批用于治疗肺部cGVHD的靶向药物。

（五）其他治疗

1. 间充质干细胞（mesenchymal stem cells, MSC）：MSC不仅具有再生能力，还具有免疫调节和修复组织的能力。MSC具有两大特性：①抑制T淋巴细胞介导的免疫反应、使机体产生免疫耐受；②抑制中性粒细胞相关的炎症反应。MSC由于免疫原性低，可以逃避细胞毒性Tc、NK细胞等免疫细胞的攻击，从而避免免疫反应导致的组织损伤排斥反应。MSC可改善HSCT后肺部cGVHD患者的肺功能，已得到广泛应用[40]。多项研究均提示MSC具有一定的临床应用价值[41]–[43]。然而，目前的研究数据显示：MSC对于重度肺部cGVHD-BOS的治疗效果欠佳。MSC的长期疗效和安全性仍需在更大规模的随机对照试验中进一步研究。

2. 体外光分离置换法（ECP）：ECP最初被用于治疗皮肤T细胞淋巴瘤及其他实体器官移植后的排斥反应。ECP主要治疗过程是预先对患者以8-甲氧基补骨脂素（8-MOP）进行预处理，然后抽取患者的全血，分离白细胞并以紫外线A照射而激活8-MOP，最后回输白细胞入患者体内。因8-MOP具有强烈的光敏活性，易被长效紫外线激活而产生光毒作用，能使细胞DNA合成及细胞分裂受到抑制、导致细胞凋亡，从而达到抑制排斥反应的目的。已有研究证明ECP在治疗aGVHD和cGVHD方面是有效的，可提高部分HSCT后BOS患者的生存率但目前尚缺乏大型RCT研究进一步验证[36]。

（六）肺移植

传统的理念认为肺移植的受体人群仅仅来自于原发性肺部疾病导致的呼吸衰竭。实际上，针对这类恶性血液病接受了HSCT，但由于肺部cGVHD导致呼吸衰竭而内科治疗无效时，可以通过肺移植挽救生命。肺移植推荐用于肺功能持续恶化且不伴有其他部位严重活动性GVHD。HSCT后晚期BOS患者接受肺移植的临床疗效方面，前期国际上观察性研究结果显示：肺移植可以挽救BOS患者的性命，延长其生存期[44]。国内国家医学呼吸中心针对肺部cGVHD（包括BOS和ILD）患者因呼吸衰竭接受肺移植的疗效进行了临床分析，在国内首次报道了HSCT患者肺移植术后74个月而具有良好生活质量[6]。这表明，肺移植作为一项补救治疗措施，其疗效逐渐得到肯定。

六、展望

肺部cGVHD是HSCT后的一个重要肺部并发症，严重影响患者的生存质量及预后。针对肺部cGVHD，无论是BOS还是ILD，一旦发生，肺功能就会进行性下降，尤其是ILD，肺功能下降速度更快。诊断方面，针对HSCT后应该从早期就关注肺功能，密切的监测肺功能，做到早期诊断早期干预，而基础研究方面需要关注cGVHD-BOS的发病机制并找出相应的生物标志物，协助指导临床早期干预，从而预防疾病的发生和发展。治疗方面，目前临床使用的药物只能改善症状、稳定肺功能或延缓肺功能的下降速度，并不能逆转肺功能。因此，在病理生理学机制研究的基础之上研发特异性的靶向药物，可望改善患者的生活质量和长期预后。
